# S. Rao Spiral Suturing (SRSS) of Lower Uterine Segment: An Innovative Hemostatic Technique in the Management of Placenta Previa and Accreta Spectrum

**DOI:** 10.12669/pjms.40.3.7747

**Published:** 2024

**Authors:** Shahid Irshad Rao, Uzma Shaheen, Syeda Husna Hasan

**Affiliations:** 1 Shahid Irshad Rao, FCPS Dept of Obs & Gynae Unit II, Nishtar Medical College & Hospital, Multan, Pakistan; 2 Uzma Shaheen, FCPS Dept of Obs & Gynae Unit II, Nishtar Medical College & Hospital, Multan, Pakistan; 3 Syeda Husna Hasan, FCPS Dept of Obs & Gynae Unit II, Nishtar Medical College & Hospital, Multan, Pakistan

**Keywords:** Placenta Previa Accreta, Spiral Suturing, Lower Uterine Segment

## Abstract

**Objective:**

The study aimed to demonstrate the efficacy and safety of an innovative hemostatic technique in managing Placenta Previa and Accreta Spectrum by S. Rao Spiral Suturing (SRSS) of a lower uterine segment.

**Method:**

In this retrospective study conducted at Department of Obstetrics & Gynecology Unit-II of Nishtar Medical University, Multan between December 2018 to January 2021, one hundred and thirty consenting patients’ clinical records were reviewed with major degree placenta previa/placenta accrete spectrum, either operated electively or presented in an emergency, with or without a history of previous cesarean section. The enrolled patients underwent SRSS, procedure’s efficacy and safety were measured by the number of obstetrical hysterectomies, the time required for the procedure, estimated blood loss, blood transfusion volume, need for any other hemostatic technique, bladder trauma, pelvic infection, scar site hematoma or abscess, sepsis, duration of hospital stay and maternal mortality.

**Results:**

Out of 130 patients, 17(12.6%) had Placenta Accreta, 86(66.3%) Increta, and 27(21%) Percreta. The Placenta location was anterior dominant in 102(78.4%) cases and posterior in 17(8.4%). Of the patients who underwent surgery, only two required obstetrical hysterectomy due to uncontrolled bleeding. The procedure took three to five minutes in 127 patients and five to seven minutes in three patients. Regarding intraoperative blood transfusion, 54.6% of patients were transfused 1000-2000 ml blood, and 5.38% required > 3000 ml. No blood transfusion was required postoperatively in any patient. Postpartum hemorrhage, infection, fever, and sepsis were not observed in any patient postoperatively. None of the patients suffered bladder injury. All patients were discharged as per routine.

**Conclusion:**

SRSS is an innovative, safe, effective, and simple suturing technique for patients with Placenta Previa and Accreta spectrum.

## INTRODUCTION

Major obstetrical hemorrhage is one of the leading causes of maternal mortality and morbidity globally. Maternal fatality associated with an increased incidence of postpartum hemorrhage and obstetrical hysterectomies in patients with major degree placenta previa/placenta previa spectrum has become very challenging to every obstetrician. In systemic reviews, the prevalence of placenta previa is approximately four per 1000 births but varies worldwide.^1^ Women with placenta previa, particularly those with previous cesarean deliveries and placenta accreta, are more likely to experience serious postpartum hemorrhage (PPH).^2^

In Pakistan, the incidence of placenta previa has been reported between 0.51%-3.5%,^3^ it has become challenging for every obstetrician in the last few years. The rate of maternal mortality due to placenta previa is 0.03%.^4^ Patients with placenta previa and a previous cesarean delivery frequently suffer from massive hemorrhage during childbirth. Hemostasis in the cesarean section of such cases can be achieved by post-placental separation suturing of bleeds. Various medical and surgical techniques, like uterotonic agents, ergot alkaloid derivatives, oxytocin, and bimanual uterine compression, control non-traumatic uterine bleeding^5^, the vascular ligation, intrauterine balloon tamponade, and uterine compression suture with B-lynch are carried out to reduce the amount of blood loss during cesarean delivery.^6^ Apart from peripartum hysterectomy, there are only a few options for controlling intractable bleeding when massive bleeding occurs. If the usual techniques fail to stop bleeding, conservative surgical procedures like ovarian, uterine, or internal iliac artery ligation are carried out.

Despite traditional compression sutures/arterial ligation, the persistence of bleeding occurs in many cases, and the patients ultimately end up in hysterectomy, which on the other hand, has a high morbidity rate and can result in negative outcomes such as the end of her reproductive ability, premature ovarian failure, physical, social and psychological trauma.^7^ The prevalence of hysterectomy following a caesarian delivery from placenta previa is around 5.3%.^8^ Perinatal mortality is 3 to 4 times higher in placenta previa than in complication-free pregnancies.

During cesarean section for placenta previa, a combined hemostatic technique consisting of transitory clamping of the ovarian artery bilaterally, intrauterine Bakri balloon tamponade, and bilateral uterine artery ligation are regarded as safe alternative methods in comparison to conventional procedures.^9^ Patients with placenta previa (total) and a previous cesarean section frequently experience massive hemorrhage during childbirth.^10^ Nonetheless, hemostasis may not be achieved because of pregnancy-related anatomical alterations, especially in the lower uterine segment (LUS), hemostasis may not be achieved.^11^

Owing to the conventional technique’s shortcomings, this study proposed a new innovative concept in which hemostasis is accomplished by direct suturing of a lower uterine segment up to the margin of the ring of internal OS rather than using indirect compression sutures for controlling intraoperative and postoperative bleeding.

## METHODS

### Study design & population:

This study was conducted at the Department of Obstetrics & Gynecology Unit II of Nishtar Medical University Multan. The clinical records of one hundred and thirty patients with major degree placenta previa/placenta accrete spectrum, either operated electively or presented in an emergency, and with or without a history of previous cesarean section, between December 2018 to January 2021, were reviewed retrospectively.

Planned cesarean sections were performed at 36-38 weeks gestation, and patients presenting in emergencies were mostly at 33-36 weeks gestation. Details of the procedure, risk of proceeding to obstetrical hysterectomy, risk of massive hemorrhage necessitating blood transfusion, and risk of bladder injury were explained.

### Ethical Approval:

It was obtained from Institutional Ethical Review Board (Ref#18677; Dated 25-09-2021).

### Diagnostic criteria:

At recruitment, placental localization, grading of placenta previa, and depth of placental invasion were determined by transvaginal sonography by using 2D greyscale. To reduce intraobserver bias, only two operators performed the ultrasound. The placenta lying within 20 mm of internal OS was labeled as placenta previa, and the placental site was classified based on location into anterior, posterior, right lateral, and left lateral.^12^ For placental invasion, unified ultrasound descriptors given in FIGO consensus guidelines were used.^13^

### Surgical Technique:

**Fig.1:**
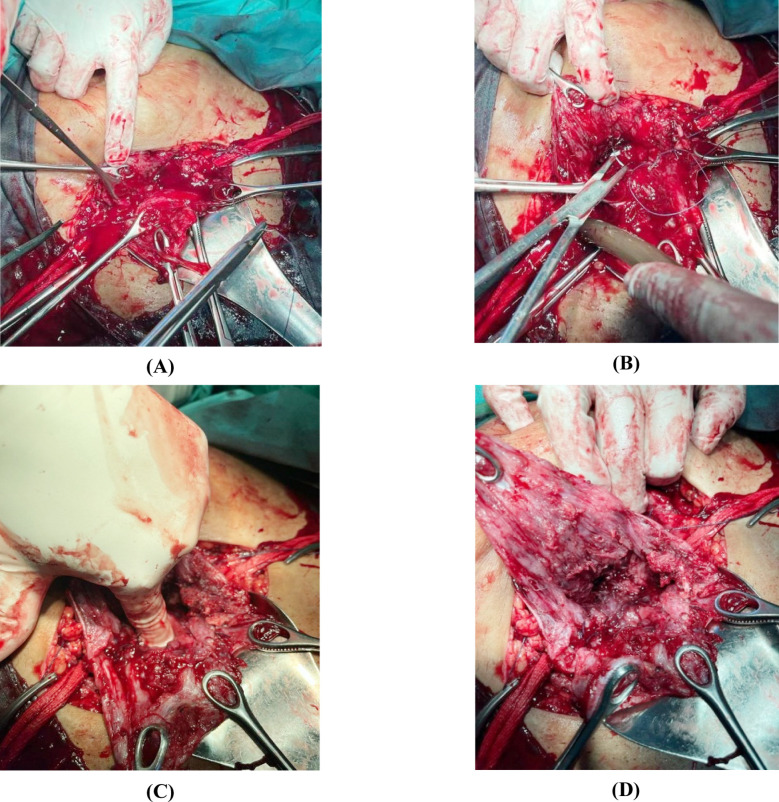
S. Rao Spiral Suturing (SRSS) of Lower Uterine Segment a.Identification of Margins of internal cervical Os, followed by the application of the S.Rao Spiral Suturing (SRSS) technique b.Suturing started from the left corner of the uterine incision, using 40mm round body needle Vicryl 1 (Ethicon) taking a partial thickness bite of 2-3cm in the lower uterine segment and another bite taken from the margin of the ring of internal cervical os and then first knot was secured. Similar partial thickness sutures were taken continuously at a distance of 1-1.5cm till the right corner of the uterine incision and second knot was secured. c.Reassuring Patency of Internal Os. d.Application of the Suture is completed.

S. Rao Spiral Suturing (SRSS) of the Lower Uterine Segment is shown in Fig.1. At the time of the cesarean section, a Pfannenstiel incision was given. In the case of adhesions found because of previous surgeries, adhesiolysis was done. The bladder was reflected down from the probable site of the uterine incision to avoid bladder trauma and delay in securing major bleeding from the placental bed in cases where the placental attachment was anterior. A transverse incision was given at the lower uterine segment (LUS). Immediately upon delivery of the baby, 10 IU of oxytocin was given intravenously. Almost 70-75% of placentas were morbidly adherent to the previous scar. After delivery of the fetus and placenta, LUS and the placental bed were visualized.

Any remnants of placental tissue were removed by blunt and sharp dissections. There is no need for exteriorization of the uterus. Sponge holder forceps held the right and left angles of the uterine incision. Internal cervical os was identified with one hand’s index and middle finger, and margins of the internal os were held with a sponge holder forceps anteriorly and posteriorly. On the exposed inner surface of the lower uterine segment (LUS), suturing was started from the left corner of the uterine incision using a 40-millimeter round body needle of polyglactin 910 suture (Vicryl No.1 Ethicon) taking a bite of about two to three cm through the half thickness of tissue of lower uterine segment (full thickness bite in cases of very thinned LUS) the other bite taken from the margin of internal os ring from inner to the outer side, a suture was tied, and 1st knot secured causing squeezing of LUS. Onwards similar sutures were taken continuously at a distance of 1-1.5 cm till it reached the right corner, and 2^nd^ knot was secured here. During suturing, we made sure patency of internal os. This continuous spiral suture controlled hemostasis by a continuous pull on sutures leading to squeezing and compression of LUS and arresting all bleeding points at the placental bed.

In cases where the placenta was attached posteriorly similar technique was used. The suturing was started from left to the right corner, taking 1^st^ bite from the posterior margin of the ring of internal os and going up to the highest point of the bleeding site in the posterior wall of the myometrium, taking a bite of 2cm, securing 1^st^ knot, taking continuous sutures at a distance of 1-1.5 cm till right corner reached where 2^nd^ knot secured. If any other bleeding point was visible, it was secured by an interrupted suture. This procedure of spiral suture is completed in three to five minutes. Then the uterine incision is closed in a double layer.

### Data Collection and follow-up assessments:

Demographic details and risk factors include maternal age, parity, number. of previous cesarean sections, gestation duration, assisted conception, previous placenta previa history, myomectomy, endometrial curettage, uterine septum resection, and endometriosis history were recorded.

Pre and postoperative blood loss were measured, and the difference was determined using the gravimetric method^14^. Blood loss on surfaces was estimated at 50 cm = 500 ml, 75 cm =1000 ml, and 100 cm =1500 ml. Blood clot measurement fist size = 50 gm. The need for blood transfusion was determined by estimated blood loss intraoperatively and a drop in hemoglobin concentration postoperatively.

All the patients were discharged on the day 3^rd^ of the surgery; none required an extra hospital stay. Postpartum hemorrhage was not observed in any patients. During their hospital stay, patients were observed for vital signs, any signs of infection, or sepsis. Patients were called for two follow-up visits, 1^st^ at after one week and 2^nd^ at six weeks. Patients were monitored for bleeding per vaginum, pelvic infection, endometritis, hematoma, and resumption of normal activity.

The efficacy and safety of the procedure were measured by the number of obstetrical hysterectomies required, estimated blood loss, blood transfusion volume, need for any other hemostatic technique, the time required for the procedure, bladder trauma, pelvic infection, scar site hematoma or abscess, sepsis, duration of hospital stay and maternal mortality.

### Data Analysis:

SPSS version 22.0 was used for data analysis. The qualitative data were presented as frequency and percentages, while quantitative data were expressed as mean and standard deviation.

## RESULTS

A total of 130 placenta previa and accrete patients were included in this study, with a mean age ranging from 25 to 40 years (mean 32.28 ± 4.21 years). The mean gestational age was 35.19 ± 1.81 weeks; 60% of patients were found to have gestational age between 34-36 weeks. One hundred and twenty-seven (97.69%) of the 130 patients had prior cesarean deliveries, and most (46.9%) had two C-sections. In 78.4% of patients, the placenta was found anteriorly, 13.07% had a posterior attachment of the placenta, while it was low-lying in 8.46% of the cases. Placenta increta was found in 66.3% of patients, percreta in 21.1%, and accreta in 12.6%.

Intra-operatively this procedure took three to five minutes in 127 patients and five to seven minutes in three patients. About 1000-2000 ml blood was transfused intraoperatively in 54.6% of patients. None of the patients suffered bladder injury. No patient required an extra hospital stay, and all were discharged within three days. Only two patients underwent obstetrical hysterectomies due to uncontrolled bleeding. No extra hemostatic technique was applied. Blood transfusion was not needed postoperatively in any patient. Postpartum hemorrhage, infection, fever, and sepsis were not observed in any patient postoperatively (Table-II).

**Table-I:** Demographics of patients included in the study (n=130).

Variables		N(%)
Age	25-30 years	35(27)
	30-35 years	52(40)
	35-40 years	43(33)
Gestational Age	34-36 weeks	78(60)
	36-38 weeks	46(35)
	38-40 weeks	06(05)
No. of Previous C-sections	None	03(2.3)
	One	25(19.3)
	Two	61(46.9)
	Three	32(24.6)
	Four	09(6.9)
Placental Location Placenta praevia (central)	Anterior dominant	102(78.4)
	Posterior	17(13.07)
	Low-lying (anterior)	11(8.46)
Placenta Accreta Spectrum (PAS)	Creta	17(12.6)
	Increta	86(66.3)
	Percreta (Grade 3a)	27(21.1)

**Table-II:** Intra-operative and postoperative findings of study patients.

Variables		N(%)
Time duration of the procedure	3-5 min	127(97.69)
	5-7 min	03(2.31)
Intra-operative blood loss	< 1000 ml	52(40.00)
	1000-2000 ml	71(54.62)
	> 2000 ml	07(5.38)
Blood transfusion	1000-2000 ml	71(54.62)
	> 2000 ml	07(5.38)
Duration of stay in hospital	1-3 days	128(98.46)
	3-4 days	02(1.54)
Hysterectomies	Yes	02(1.54)
	No	128(98.46)

## DISCUSSION

Advances in medical technology, interventional therapy, and pharmacotherapy have demonstrated significant benefits for the management of post postpartum hemorrhage. On the other hand, a surgical suture is the efficacious hemostatic method for managing postpartum hemorrhage, as demonstrated by the SRSS technique in our study and other studies.^15^-^17^ This study proposed an innovative technique in response to anatomical alterations and the unsatisfactory hemostasis effects of conventional external compression techniques. It entailed suturing the bleeding site directly rather than using indirect compression sutures to provide better hemostasis. It also suggested using spiral sutures in LUS to manage intraoperative and postoperative bleeding by directly suturing the bleeding area. Spiral sutures lead to a very minimal number of hysterectomies compared to other sutures. In a comprehensive evaluation of 10 studies on the therapy of placenta previa accreta at birth conducted by Jauniaux et al and Bhide et al.^18^, it was discovered that 208 (89.7%) cases involved elective or urgent cesarean hysterectomy.

The common external compression suture procedures work by limiting outlying tissues to control severe bleeding. While in the recent study with spiral sutures, only two patients underwent a direct obstetrical hysterectomy. No traditional or classical hemostatic techniques were used in our study. In a similar study, it was reported that their vertical parallel compression suturing had a higher success rate in hemostasis and uterus preservation in cases of placenta previa and accreta than in surgeries without this technique.^19^ However, the technique was found ineffective in cases of placenta increta. In contrast, our newly developed spiral suturing technique could easily be performed in all placenta previa.

This suturing procedure returns the uterus to its normal anatomical morphology by immediately attaining hemostasis. Only seven patients in the current investigation experienced excessive blood loss (> 3000 mL), possibly because the spiral suturing was used alone. In a similar study, no patient experienced excessive blood loss when treated with the combination of spiral sutures and aortic balloon occlusion. Although aortic rupture, abdominal aorta dissection, reperfusion injury, and the inability to deflate or extract the balloon through the sheath are some potential dangers of balloon inflation.^20^

The suture’s simplicity, lack of need for major treatments or pricey equipment, and increased multidisciplinary involvement make it unique. This innovative spiral suturing involves suturing in a field full of blood which demands the operator to be swift and in self-control. The severity of the hemorrhage is typically controlled after the first three to four serial sutures, allowing the remainder of the treatment to be completed quite quickly. The problem can be avoided in people with hematoma development by shortening the space between the sutures. Most isolated hematomas are minor and easily treated with additional hemostatic sutures.

### Limitations:

It includes small sample size was the major limitation of the study. An adequately high-powered randomized control trial would be required to enhance this surgical technique’s validity and assess its suitability for placenta percreta beyond Grade 3a.

## CONCLUSION

According to the study findings, S. Rao Spiral Suturing (SRSS) was an innovative technique that caused only obstetrical hysterectomy in two patients without significant immediate or delayed complications such as the need for blood transfusion, postpartum hemorrhage, infection, fever, or sepsis.
